# Rheostat Coordination of Latent Kaposi Sarcoma-Associated Herpesvirus RNA Expression in Single Cells

**DOI:** 10.1128/JVI.00032-21

**Published:** 2021-08-10

**Authors:** Nicole C. Rondeau, JJ L. Miranda

**Affiliations:** a Department of Biology, Barnard College, Columbia University, New York, New York, USA; University of Arizona

**Keywords:** Kaposi’s sarcoma-associated herpesvirus, genomics, herpesviruses, latent infection

## LETTER

We detected precise coordination of RNA levels between two latent genes of the Kaposi sarcoma-associated herpesvirus (KSHV) using single-cell RNA sequencing. LANA and vIL6 are expressed during latency by different promoters on remote regions of the episome. We observe that LANA and vIL6 are quantitatively coexpressed across several orders of magnitude like a rheostat in individual cells. Reminiscent of transcriptional noise, this variability exists in an unstimulated population.

We measured viral and human RNA expression (1) in the BC-1 primary effusion lymphoma cell line (2) at single-cell resolution using Chromium 10x Gel bead in EMulsion microfluidic technology (3). Here, we analyze the quantitative relationships between KSHV transcript levels using the previously published data sets (1). Among viral genes, LANA and vIL6 were detected in the most cells. Using the mean nonzero counts per cell as a metric, LANA and vIL6 were expressed among the top 1% and 10%, respectively, of all human and viral genes (data not shown).

**FIG 1:**
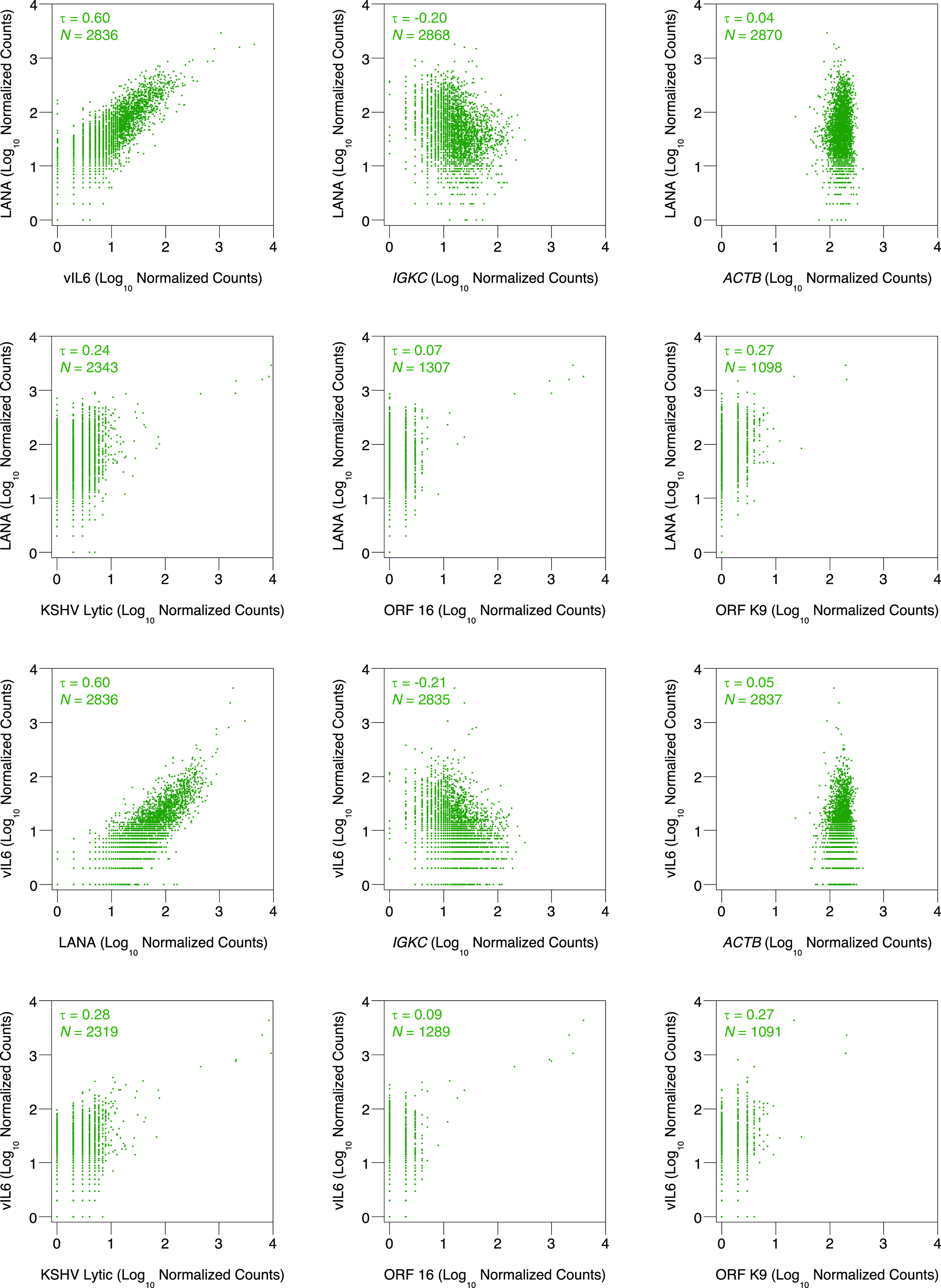
Expression correlation of KSHV and human RNA levels in individual BC-1 cells as measured by single-cell RNA sequencing. Log_10_ values of normalized counts for the latent KSHV transcripts LANA and vIL6 are plotted against each other, the lytic KSHV transcripts ORF 16 and ORF K9, the union of all lytic KSHV transcripts, or the host transcripts *IGKC* and *ACTB*. Each dot represents a single cell. Cells with zero counts for either component plotted have been omitted to minimize the effects of dropout events. Data points with identical values overlap. Correlation coefficients are shown as Kendall’s *τ* with a *P* value of <0.05. *N* denotes the number of cells depicted in each graph. Data shown represent one biological replicate and reproduce similarly in a second biological replicate.

LANA and vIL6 RNA levels strikingly correlate across ∼3 orders of magnitude (Fig. 1). This occurs even though different promoters control LANA (4) and vIL6 (5) expression. Our counting protocol (1) only assigns reads to a single gene if that sequence does not overlap with another annotation. No confounding reads that map to both LANA and vIL6 were detected. Kendall’s correlation coefficient between LANA and vIL6 is strong (*τ* = 0.52 to 0.60) and higher than that between LANA or vIL6 and other KSHV genes (Table 1). Although lack of frequent detection may yield weak or insignificant values, the large number of cooccurrences between LANA and vIL6 is not sufficient to explain the strong correlation. Among KSHV genes (Table 1), we observed no statistically significantly correlation (*P* > 0.05) between the statistically significant correlation coefficients and the number of simultaneous detections (data not shown). We also measured relationships with host housekeeping genes as a control. *IGKC* displays the largest coefficient of variation among transcripts with expression counts similar to LANA and vIL6 (data not shown). *ACTB* is a frequently used reference with lower variance between cells (data not shown). Neither LANA nor vIL6 shows strong correlation with *IGKC* or *ACTB* (*τ* = −0.24 to 0.08) (Table 1).

**TABLE 1:** Correlation of RNA expression between KSHV LANA or vIL6 and viral or human transcripts in single BC-1 cells

Transcript	Replicate 1^*a*^		Replicate 2^*a*^	
	KSHV LANA	KSHV vIL6	KSHV LANA	KSHV vIL6
KSHV^*b*^				
ORF K2 vIL6	0.60^*c*^	1.00	0.52	1.00
ORF 2	NS^*d*^	0.17	NS	NS
ORF K4	0.18	0.26	0.26	0.29
ORF K5	0.29	0.39	0.34	0.37
PAN	0.15	0.20	0.24	0.29
ORF 16	0.07	0.09	0.14	0.13
ORF 45	NS	0.14	0.24	0.27
ORF 54	NS	0.12	0.15	0.16
ORF K9	0.27	0.27	0.08	0.12
ORF K10	NS	NS	0.19	0.20
ORF 73 LANA	1.00	0.60	1.00	0.52
ORF 75	NS	NS	NS	NS
Human^*e*^				
*IGKC*	−0.20	−0.21	−0.21	−0.24
*ACTB*	0.04	0.05	0.06	0.08
EBV^*b*^^,^^*f*^				
EBER-2	NS	NS	NS	NS
A73, BARF0, RPMS1	NS	NS	NS	NS

a Independent biological replicates.

b Only correlations with viral transcripts present in >5% of cells in at least one replicate are shown.

c Values reported as Kendall’s *τ* with *P* values of <0.05 using the cor.test function as packaged in R.

d NS, not significant with *P* values of >0.05.

e Only correlations with select human genes are shown.

f EBV, Epstein-Barr virus.

The dynamic coordination of LANA and vIL6 occurs in the absence of lytic replication. LANA can be expressed by a lytic promoter (6), and vIL6 is upregulated during the lytic phase (7), so we checked if the gradient of LANA and vIL6 expression reflected lytic replication. LANA and vIL6 correlate less strongly with the lytic gene ORF 16 or ORF K9 (*τ* = 0.07 to 0.27) (Table 1) than with each other (*τ* = 0.52 to 0.60). Only a few cells contain high ORF 16 or ORF K9 RNA expression, indicative of reactivation (Fig. 1). On the other hand, most cells contain low ORF 16 or ORF K9 levels indicative of latency. The gradient of LANA and vIL6 RNA expression still spans ∼2 to 3 orders of magnitude in latent cells. The same trend is seen when comparing LANA or vIL6 RNA expression with the sum of all lytic KSHV gene counts as a measure of reactivation (Fig. 1). This behavior contrasts with the coordinated continuum of human cytomegalovirus lytic gene expression (8). Noncanonical biphasic models of herpesvirus reactivation propose that latency is not a restricted static state but instead consists of dynamic activity that approaches a threshold necessary for commitment to virion production (9). A rheostat of latent RNA may represent the first phase prior to the canonical cascade of lytic gene expression. The interaction between LANA and vIL6 themselves is indeed coordinated across a spectrum of latency.

We note that coordination of LANA and vIL6 RNA expression is observed without delivered external stimulus. Heterogeneity in RNA levels of single genes to generate a diverse population from genetically identical individuals is prevalent in microbes (10). Examples include bacterial transition to competence (11) and yeast growth under heat stress (12). With KSHV, two genes vary together in a concerted fashion to generate a heterogeneous population. We are curious if multigene rheostats similar to this example also exist in other cell lines with KSHV, other latent viruses, or other microbes.

KSHV latency occurs on a spectrum, but what does this rheostat measure or control? We hope that our observations of LANA and vIL6 precise coordination fuel further study. The molecular basis and phenotypic consequence of concerted stochastic latent RNA expression remain to be explored. We nonetheless suggest that the definition of viral latency be continually revisited.
